# Some Aspects of Medical Science

**Published:** 1894-08-11

**Authors:** 


### Some Aspects of Medical Science.

In this our last discussion on the relative sexual differences in acuteness and precision of sensory perceptions we propose dealing with a comparison between the eyesight of the two sexes. And not alone for the sake of the mere comparison has this matter been entered into, but an important question of the hour is here involved, and that is regarding the fundamental fitness of women for engaging on equal terms in the same occupations as a man may, and without such comparison how can this, even in theory, be settled? Mr. Havelock Ellis has collected many valuable data together on this comparison, a work which, from its abstruse character, involved a considerable outlay of time and thought, and we feel much indebted to our authority for all which he has brought to bear upon the subject.

Blindness in this country, if we turn to the census of 1891, is found to be far more common among males than among females, at all ages up to sixty-five, after which period, as our authority suggests, the preponderance of blind women is due in all probability to the greater length of women's lives. Mr. Brudenell Carter, who has made this an especial subject of research, analysed ten thousand cases of diseases of the eyes, and gives us the following tabular matter as a result:—

| | Males. | Females. | Total. |
|---|---|---|---|
| Emmetropia or normal eyesight ... | 2,123 | 2,318 | 4,441 |
| Short sight or myopia, including simple and compound myopic-astigmatism, or irregularity of eyeball ... | 1,464 | 1,684 | 3,148 |
| Long sight, or hypermetropia, including simple and compound hypermetropic astigmatism ... ... ... | 995 | 1,328 | 2,323 |
| Mixed astigmatism ... ... ... | 39 | 49 | 88 |
| Totals ... ... ... ... | 4,621 | 5,379 | 10,000 |

Therefore, among Mr. Carter's patients there has been shown a marked and decided preponderance of women and girls. In his contribution in reference to the matter, the doctor refers "to the greater sensitiveness of the female sex, to the more sedulous employment of their eyes over a variety of sedentary occupations, and to their weaker muscles, which are less able, as a rule, than those of men, to maintain prolonged efforts of accommodation or of convergence." At the same time Mr. Brudenell Carter points out that the preponderance of cases of defective eyesight in the case of women is not to be explained by special proclivity on the part of women to any single form of eye disease.

All over Europe the eyesight in boys and girls' schools has been examined for this purpose, and again the results are in accordance with Mr. Carter's, shortsightedness being decidedly more common among girls than among boys. In Sweden, for instance, Professor Axel Key discovered, after assiduous labours in the same direction, that among 11,000 boys the shortsightedness ranged from 6 per cent. at the age of 11, to 37·3 per cent. at the age of 19; whilst amongst the girls in these Swedish schools the defect was observable, ranging from 21·4 per cent. at the age of 10 to 50 per cent. and more at the age of 20.

In America the tests applied by Dr. West showed the same result; but whilst the percentage of defective eyes certainly exceeded in the case of the girls, the defects shown in the boys' eyesight were of a decidedly graver nature. And some of us may remember the result of Dr. F. Warner's experiments—very considerable ones, involving an examination of no less a number than 60,000 school children. His results proved themselves quite in accordance with the American's.

So much for the principal comparisons which we have at hand to offer on the prevalence of eye defects. We will next turn our attention to a more specialised form of the same subject—to the relative keenness of eyesight in man and woman. Here the field of observation is of a narrower character, and we have fewer data to go upon.

Galton's tests, which were applied at Bath on members of the British Association, showed curiously enough that masculine sight was better with the right eye, feminine with the left. But beyond this fact little sexual difference was really noticeable. At the Health Exhibition Laboratory Mr. Galton stated that men were generally found to be slightly superior to women in keenness of vision. Jacobs and Spielman, Mr. Ellis reminds us, found that the English Jewess is decidedly superior to the English Jew in keenness of sight, both in the average and in the maximum and minimum.

At Wisconsin University some experiments of this nature were conducted under the direction of Professor Jastrow,* who caused the relative sexual strength of vision to be tested by noting the smallest letter readable at 25 feet through one and through two thicknesses of common cheese cloths; the result in dioptrics being—through one thickness—24·7 for the men and 19·0 for the women; through two thicknesses, 45·0 for the men and 4·20 for the women. At the same time acuteness of vision was tested in other ways—such as a series of lines 1 mm. wide, and separated by spaces of 1 mm., and irregularly arranged dots; but in all Professor Jastrow's experiments in this direction the men were shown to have a very slight advantage in eyesight.

Taking all this evidence as a whole, Mr. Havelock Ellis points out that we may fairly well conclude that in most, if not all, civilised countries women are more liable to the slight disturbance of eyesight, due to defective accommodations, which are probably associated with "civilisation," whilst eye defects, in the case of the sterner sex, are generally of a more serious character. We have only to descend to a lower scale of beings to have a confirmation furnished us of the association of eye defects with "civilized" conditions. Motais, in a contribution to the Paris Academy of Sciences, stated that having examined the eyes of wild beasts captured after they had reached adult age, he found them normal; those captured earlier, and still more those born in captivity, were short-sighted. There is, however, no actually marked sexual difference in the strength and acuteness of vision of man and woman if we confine our tests to the healthiest classes of the community.

* "Studies," &c., *American Journal Psychology*, April, 1892.

